# Impact of constant light exposure during pregnancy on skin of neonatal New Zealand rabbits: structural and ultrastructural study

**DOI:** 10.1590/1414-431X202010722

**Published:** 2021-04-19

**Authors:** A.G. Elsaid, N.M. Faheem

**Affiliations:** 1 Department of Physiotherapy, College of Applied Medical Sciences, Taif University, Taif, Saudi Arabia; 2 Department of Anatomy and Embryology, Faculty of Medicine, Ain Shams University, Cairo, Egypt

**Keywords:** Constant light, Skin, Neonatal, PCNA, Melatonin

## Abstract

Continuous industrial productivity and modern societies have resulted in excess artificial light. The altered circadian rhythm causes many diseases. During intrauterine life, the mother's maternal melatonin rhythm has a major role in influencing organ development. The aim of this study was to investigate the effect of maternal exposure to constant light on the structure and ultrastructure of neonatal skin. Twenty pregnant New Zealand rabbits were divided into two groups (n=10 each): control group (12-h light/dark) and constant light group (24-h light). Plasma maternal melatonin and corticosterone during pregnancy were determined. At the end of the experiment, the dorsal skin of the neonates of both groups was collected and prepared for histological, morphometric, and transmission electron microscopic study. Histological and morphometric results of skin of neonates from the constant light group revealed statistically significantly reduced epidermal thickness, decreased number of hair follicle, increased surface area of collagen, and decreased proliferating cell nuclear antigen (PCNA) positive cells. Ultrastructural examination showed wide intercellular spaces and disrupted desmosomal junctions in the epidermis. Earlier stages of hair follicles were also observed with indented shrunken nuclei, vacuolization, and swollen mitochondria. Dermal fibroblasts with dilated cisternae of rough endoplasmic reticulum containing electron-dense material were detected. Maternal melatonin was significantly reduced in the constant light group while maternal corticosterone showed no significant difference between groups. Therefore, normal maternal circadian rhythm is a key factor for the integrity of neonatal skin structure.

## Introduction

The skin is the largest organ of the body; it occupies about one-sixth of total adult body weight. It has many vital functions, such as regulation of body temperature, protection against external physical, chemical, and biological stimuli, and prevention of excess water loss from the body (1). The hair follicle is a characteristic feature of humans and other mammals. Hair in humans serves many functions such as protection against external stimuli. The hair also has an important psychosocial role in society. Hair follicle number and distribution over the body and features of each hair is established during fetal development; no extra follicles are produced after birth (2). Hair follicles and sebaceous glands are developed from neuroectodermal and mesodermal origin. They are strongly correlated to the neuroendocrine system and affected by its hormones, e.g., prolactin, melatonin, corticosterone, and thyroid hormones (3).

Several biophysical and physiological functions of skin, such as transepidermal water loss, keratinocyte proliferation, skin blood flow, and skin temperature, were found to have circadian variations (4). The skin expresses a circadian clock, which is active within keratinocytes and dermal fibroblasts (5). Circadian rhythms are developed by the suprachiasmatic nuclei (SCN) in the anterior hypothalamus. Light information is received by the retina, which transmits it to the SCN. Then, this information is passed to the rest of the body through neural or hormonal routes. Studies reported that suppression or removal of the SCN leads to arrhythmic clock gene expression in the skin; these findings can be found in the total count of the cells of the skin (6,7). The SCN controls daily rhythms in hormone concentrations such as melatonin and corticosteroids (8).

During pregnancy, melatonin of maternal origin, known by its ability to cross all physiological barriers as the placenta, influences the fetus by its rhythm. This maternal melatonin rhythm has a major role in influencing fetal development (9).

However, with the need for continuous industrial productivity, the illuminated night of modern societies, and continuous patient's care in intensive care units, many workers cannot match their sleep/wake cycle to the light/dark cycle (10). Moreover, altered circadian rhythms are essential causes of many diseases such as diabetes, mental health disturbance, heart disease, and cancer (11).

The extent and quality of mother-pup interaction is a powerful determinant of offspring behavior later in life because the maternal circadian system acts as the primary entraining cue to offspring during embryonic and early neonatal life (12). Accordingly, maternal chronodisruption with the associated melatonin suppression causes many disturbances in the rodent circadian rhythm of the fetus and newborn (13). Constant light exposure during the last third of gestation leads to precocious fetal adrenal maturation with the resultant increased serum cortisol levels in the newborn immediately after birth (14). Moreover, gestational exposure to constant light impairs the development of immune tolerance of the neonatal skin (15).

New Zealand white rabbits, characterized by their intermediate size and their close phylogeny to primates, have received great attention as animal models for biomedical and pharmaceutical research that bridge the gap between rodents and the large animal models (16).

To our knowledge, no study was performed to show the effect of maternal exposure to constant light on the structure of skin of neonatal rabbits. Accordingly, the objectives of this study was to determine the impact of constant light on maternal nocturnal plasma levels of melatonin and corticosterone and to explore the structural and ultrastructural alterations in skin, as a window to the state of general health state, of neonates of constant-light-exposed mothers.

## Material and Methods

### Experimental animals

Twenty female and ten male one-year-old New Zealand white rabbits (2.5-3.0 kg each) were used in this study. They were locally bred and provided with veterinary care in the Medical Research Centre, Faculty of Medicine, Taif University (Saudi Arabia). They were housed in cages with food and water *ad libitum*.

Animal rights were ensured as determined by the rules of National Institutes of Health for the Care and Use of Laboratory Animals. All experimental protocols, including the use of animals, were approved by the Committee of Research Ethics for Laboratory Animal Care, Taif University (Approval No. 42-0052). All efforts were made to minimize suffering.

Male and female rabbits were housed separately for two weeks before proceeding with the study. Then, each male was kept in a cage with two females for the night. In the morning, pregnancy of the rabbit was detected by vaginal smear. Ten days after mating, rabbits were palpated to confirm pregnancy. All pregnant rabbits were housed individually in stainless steel cages (60×60×40 cm).

### Experimental design

Prenatally, the day of mating was considered day one of gestation while the day of delivery was considered day one of postnatal life. The pregnant rabbits were divided into two groups.

*Control group*. Ten pregnant rabbits were kept under regular laboratory lighting conditions (12-h light/dark cycle) during the same period. This was achieved by switching on the light at 6:00 a.m. and off at 18:00 p.m. The total number of the offspring was 46; each rabbit gave birth to 4-5 offspring.

*Constant light group.* Ten pregnant rabbits were placed under constant light conditions (24 h/day) by a fluorescent lamp (light intensity of 600 lux) for the whole period of pregnancy. The total number of the offspring was 27; each rabbit gave birth to 2-3 offspring.

### Biochemical studies

The blood samples were collected in the evening (8:00 p.m.) from the ear veins. For local anesthesia, Prila cream (2.5% lidocaine + 2.5% prilocaine, Avalon pharma, Saudi Arabia) was applied at the site of the venipuncture. Four blood samples were taken from each female on the 8th, 16th, 24th, and 30th day of gestation to measure melatonin and corticosterone plasma levels.

Blood was collected into ice-cold tubes containing EDTA and centrifuged at 1792 *g* for 10 min at 4°C. The plasma was stored at -20°C until assayed. Melatonin and corticosterone were measured by ELISA test kit (Glory-Science, USA) based on the instructions of the manufacturer.

### Tissue preparation for histopathological examination

After delivery, the neonates were separated from their mothers at the day of birth. Skin samples from the dorsolumbar part of the trunk of the neonates were dissected after injection of 0.1 mL of local anesthetic (2% lidocaine hydrochloride, (Pharmaceutical Solution Industries, Saudi Arabia) and samples were preserved in 10% neutral buffered formalin (Sigma-Aldrich) (for 2-5 days) and Bouin's solution (Sigma-Aldrich) (for 48 h). The skin samples were then dehydrated in ascending grades of ethanol, cleared in xylene, and impregnated with melted paraffin wax. Finally, paraffin blocks of the processed samples were prepared. Thin sections (5-μm thick) were cut and mounted on egg-coated glass slides, dried in an electrical incubator (at 37°C) for 30-60 min, and stained with hematoxylin and eosin (H&E) and Masson's trichrome technique.

### Immunohistochemical study

Paraffin sections were dewaxed, hydrated, and immersed in an antigen retrieval solution (0.01 M citrate buffer, pH 6.0) for 15 min and autoclaved at 121°C for 15 min. They were then treated with 0.3% hydrogen peroxide and protein block, followed by incubation with anti-PCNA (clone PC10; 1:200 Dako, Denmark) at 4°C overnight. The slides were rinsed three times with PBS, incubated with anti-mouse IgG secondary antibodies (Sigma-Aldrich) for 30 min at room temperature, visualized with diaminobenzidine commercial kits (Thermo Fisher Scientific, USA). PCNA appeared as brown nuclear staining.

### Transmission electron microscopic (TEM) study

Other sections of the skin of the dorsolumbar part of the trunk of the neonates were cut in small pieces of 1 mm^2^ in size and fixed in 2.5% glutaraldehyde for 24 h. Specimens were washed in 0.1 M phosphate buffer at 4°C, then post-fixed in 1% osmium tetroxide at room temperature. Specimens were dehydrated in 9 ascending grades of ethyl alcohol, and then embedded in epoxy resin. Ultrathin sections (50 nm) were cut, mounted on copper grids, and stained with uranyl acetate and lead citrate. Specimens were examined and photographed with transmission electron microscope (Jeol-Jem 1010, Japan) at the Faculty of Science, Azhar University (17).

### Morphometric analyses

The image analyzer computer system Leica Qwin 500 (England) was used to evaluate the following parameters: i) epidermal thickness: measured from the top of the horny layer to the basal membrane using H&E stain. This was performed in five non-overlapping fields from two sections each neonate, with a ×400 objective; ii) number of hair follicles/standard area using H&E stain: frame of a standard area equal to 1,443,340.6 μm^2^ were chosen from 5 different sections of each neonate, with a ×400 objective; iii) area percentage occupied by collagen fibers using Masson's trichrome stain measured in five non-overlapping fields from two different sections of each neonate, with a ×400 objective. Within the measuring frame, the area percentage was detected and masked by a binary color; and iv) percentage of PCNA immunopositive cells/field calculated by assessing the number of positively stained nuclei compared to the number of total nuclei in stratum basale per field from 5 different sections of each neonate, with a ×400 objective. All the parameters were measured in 5 randomly chosen neonate rabbits/group.

### Statistical analysis

The data are reported as means±SD. Statistical significance was determined using the unpaired *t*-test (SPSS Inc., IBM, USA). Differences were considered significant if P was less than 0.05 and highly significant if P was less than 0.001.

## Results

### Serological results

**Figure 1:**
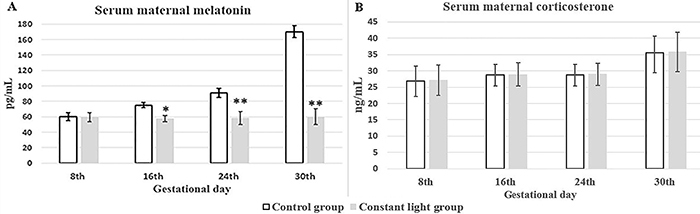
A, Means±SD of maternal nocturnal serum melatonin and (**B**) serum corticosterone among the two studied groups at the 8th, 16th, 24th, and 30th gestational day. *P<0.05, **P<0.001 *vs* control group at same gestational day (unpaired *t*-test).

The mean maternal nocturnal serum melatonin progressively increased throughout the gestation with the maximum value on the 30th gestational day in the control group, while the serum melatonin levels were nearly constant throughout the gestation in the constant light group. There was no significant (P>0.05) difference in maternal nocturnal serum melatonin between groups on the 8th gestational day. The maternal melatonin levels of the constant light group reduced significantly (P<0.05) on the 16th gestational day and more significantly (P<0.001) on the 24th and 30th gestational days compared to the control group (Figure 1A).

The serum corticosterone levels of both groups were relatively constant during gestation with a slight elevation on the 30th day of gestation. The constant light during gestation had no effect on mean maternal plasma corticosterone levels. There was no significant (P>0.05) difference in the corticosterone concentration at any gestational age between both groups (Figure 1B).

### Light microscopic results

**Figure 2:**
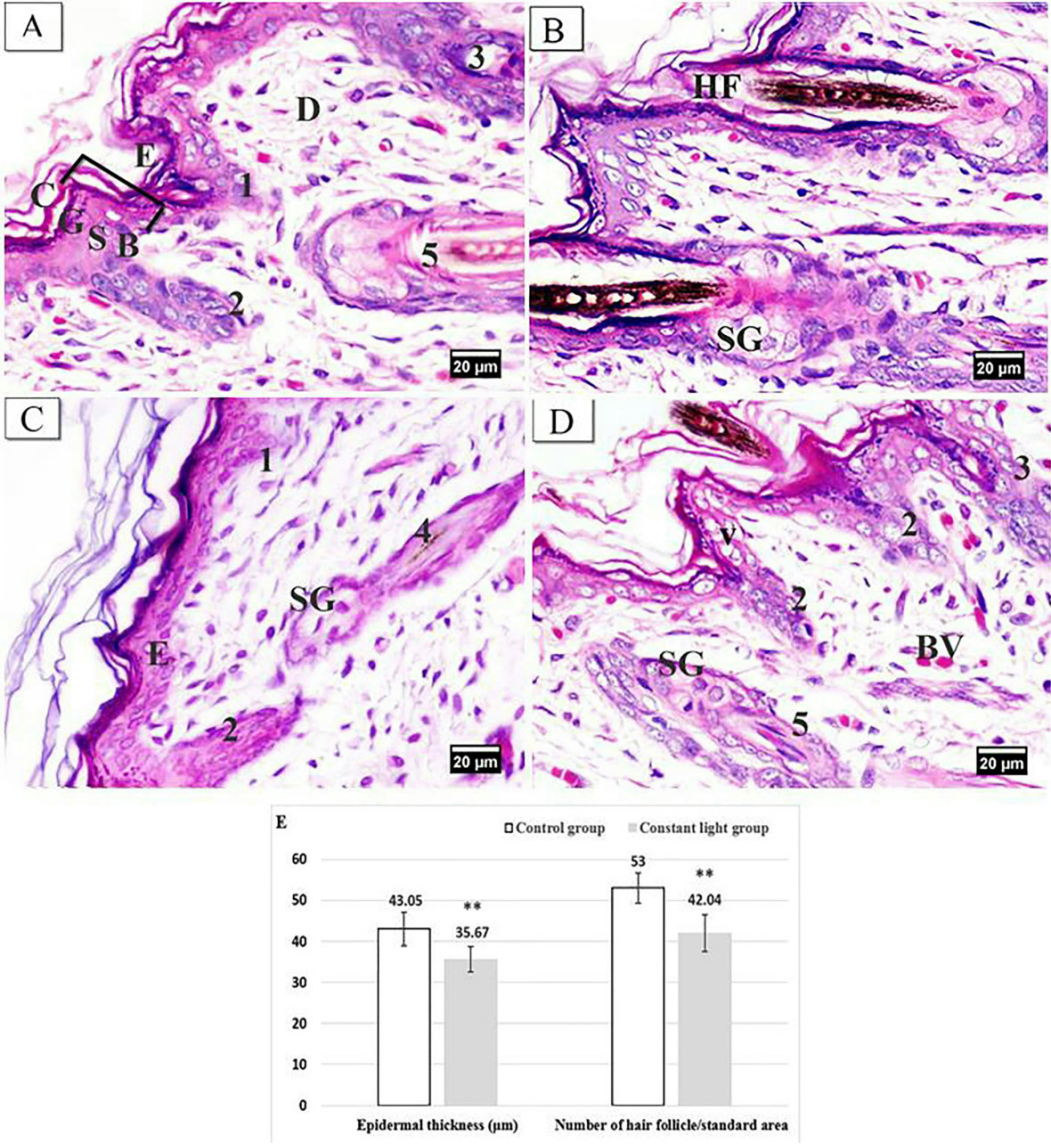
Photomicrographs of H&E-stained sections of dorsal skin of neonate rabbits (×400, scale bar 20 μm). **A** and **B**, Control group showing: **A**, epidermis (E), stratum corneum (C), stratum granulosum (G), stratum spinosum (S), stratum basale (B), dermis (D), and the different stages of hair follicle morphogenesis (stage 1, 2, 3, 5); **B**, terminal stage of the hair follicle (HF) and sebaceous gland (SG). **C** and **D**, Constant light group showing: **C**, markedly reduced epidermal thickening (E), early stages of hair follicles (1,2,4), and sebaceous gland (SG); **D**, keratinocytes shows vacuolated cytoplasm (V), congested dermal blood vessels (BV), and different stages of hair follicle morphogenesis (stage 2, 3, 5). **E**, Means±SD of epidermal thickness and number of hair follicles per standard area. **P<0.001 *vs* control group (unpaired t-test).

H&E-stained sections of dorsal skin from neonate rabbits of the control group showed normal histological architecture. The epidermis that formed the uppermost multi-layered compartment of the skin showed a keratinized stratified squamous epithelium with four distinct layers: stratum corneum, stratum granulosum, stratum spinosum, and stratum basale, which rested on the basement membrane. Different stages of hair follicle morphogenesis were detected in the dermis. The majority of the hair follicles were in the terminal stage where the hair shafts extended and protruded through the hair canal upon the surface of the epidermis and were associated with well-developed sebaceous glands in the dermis (Figure 2A and B).

H&E-stained sections of neonates of the constant light group showed markedly reduced epidermal thickening with some vacuolated keratinocytes. Most of the detected hair follicles were in early stages of development. The first stage of hair follicle showed a primary hair germ. The second stage revealed an elongated hair bud. The third stage showed an elongated column, containing radially arranged keratinocytes, intruded by dermal cells from its lower end to form the dermal papilla. Fourth and fifth stages were characterized by the presence of immature and mature, respectively, inner root sheath. The sebaceous glands appeared as small buddings from the upper third of the outer root sheath of the hair follicle in the dermis. Moreover, congested blood vessels were evident in dermis (Figure 2C and D).

Statistical comparison revealed that constant light led to a highly significant decrease (P<0.001) in the epidermal thickness and hair follicle number/standard area in the neonates of the constant light group compared to the control ones (Figure 2E).

**Figure 3:**
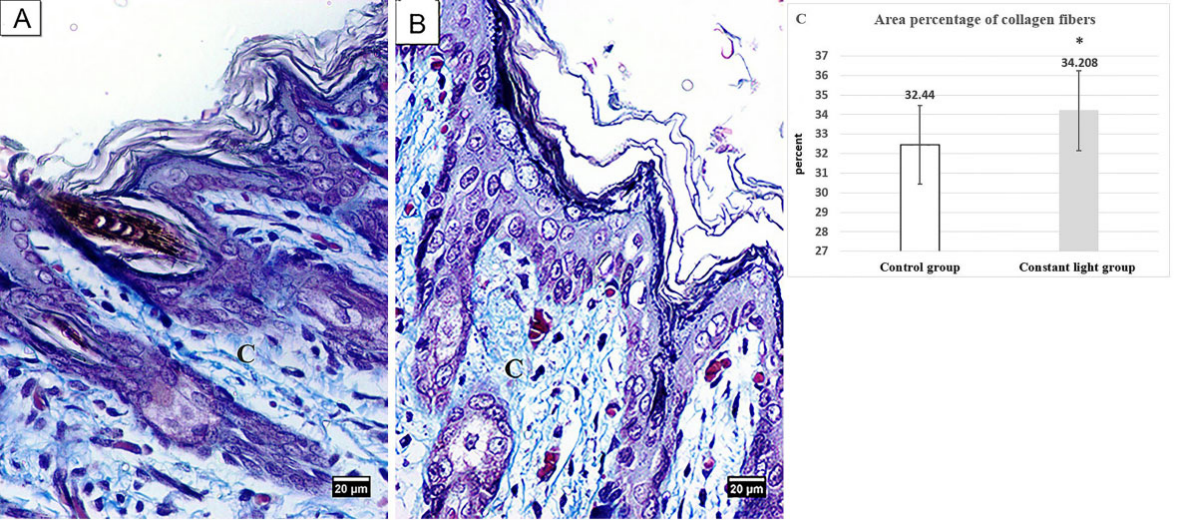
Photomicrographs of Masson's trichrome stained sections of dorsal skin of neonate rabbits (×400, scale bar 20 μm). **A**, Control group showing regularly arranged collagen fibers (C) in the dermis. **B**, Constant light group showing abundant collagen fibers (C) in the dermis. **C**, Means±SD of area percentage of collagen fibers/field. *P<0.05 *vs* control group.

Masson's trichrome-stained sections showed regularly arranged collagen fibers in the dermis of control neonates (Figure 3A). Statistical analysis revealed that area percentage of collagen fibers was significantly increased (P<0.05) in the neonates of the constant light group compared to that of the control ones (Figure 3B and C).

### Immunohistological results

**Figure 4:**
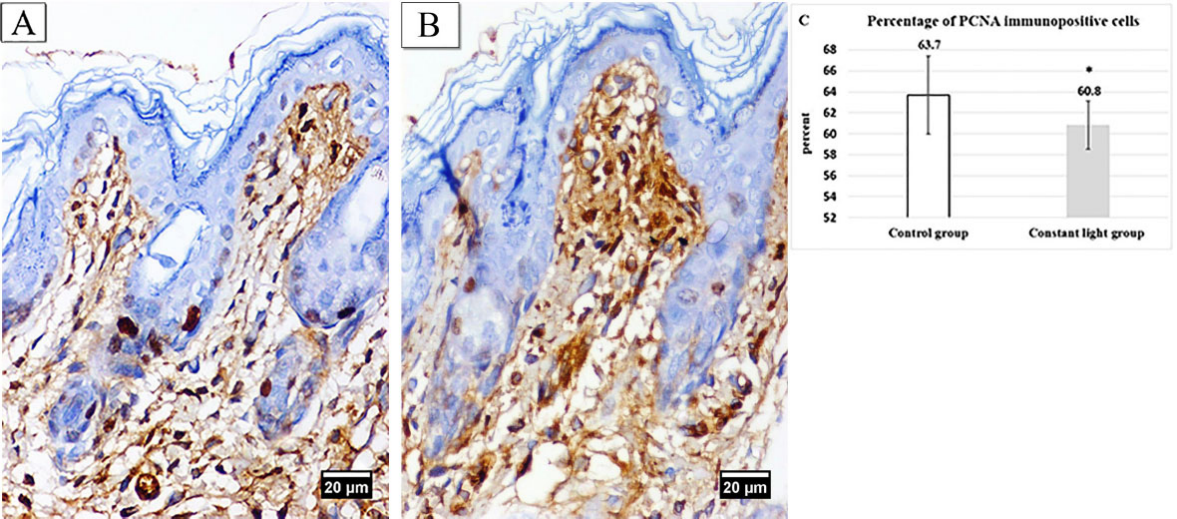
Immunohistochemical staining for the PCNA of dorsal skin of neonate rabbits (×400, scale bar 20 μm). **A**, Control group showed many positive immunoreactive nuclei of stratum basale. **B**, Constant light group showed less positive immunoreactive nuclei of stratum basale. **C**, Means±SD of percentage of PCNA immunopositive cells in stratum basale. *P<0.05 *vs* control group.

Skin sections from the control group showed positive PCNA immunoreactivity in nuclei of most cells of stratum basale, few stratum spinosum cells, and cells of outer root sheath of hair follicles in addition to fibroblast cells in dermis (Figure 4A). However, the constant light group recorded a significantly (P<0.05) lower percentage of PCNA immunopositive cells/field in stratum basale compared with the control group (Figure 4B and C).

### TEM results

**Figure 5:**
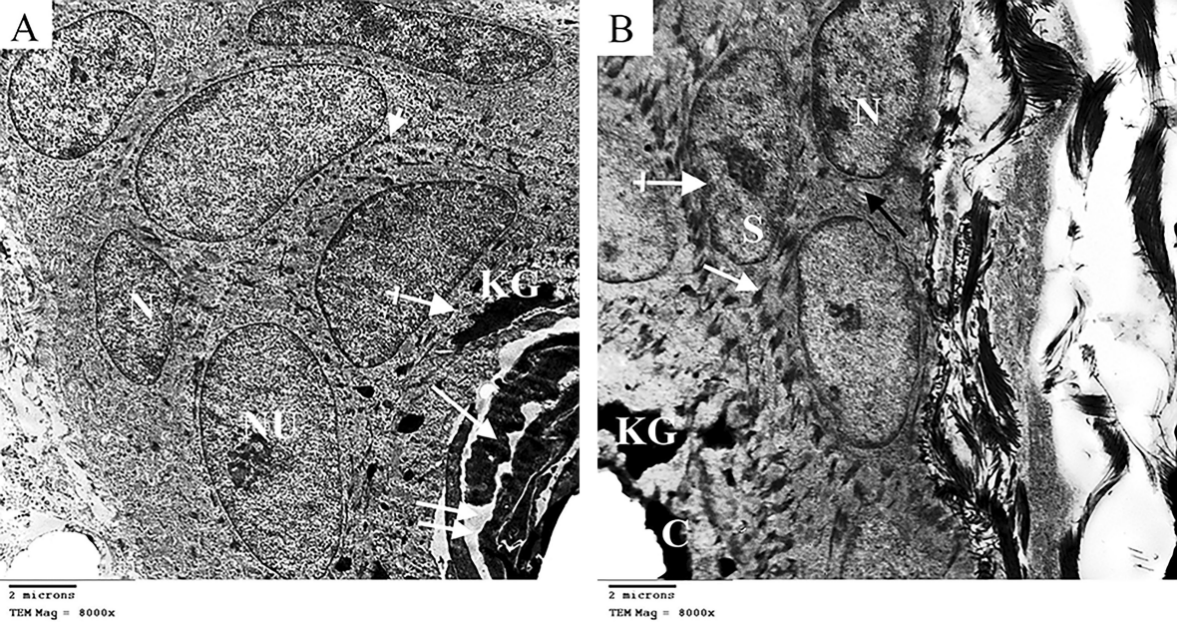
Electron micrographs of epidermis of neonate rabbits (×8000, scale bar 2 μm). **A**, Control group showing horny (arrow) and lipid layers (double arrows) of stratum corneum, stratum granulosum with its normal fibrillar content (crossed arrow) and keratohyalin granules (KG), stratum spinosum cells with euchromatic nuclei and prominent nucleoli (NU), desmosomes (arrow head), and stratum basale cells with basal oval nuclei (N). **B**, Constant light group showing thin stratum corneum (C), few keratohyalin granules (KG), flattened stratum spinosum cells (S), basal cell with heterochromatic nuclei (N), wide intercellular spaces (black arrow), desmosomal junction disruption (white arrow), and irregular nuclear membrane (crossed arrow).

The ultrastructural study of neonatal epidermis of the control group showed a well-arranged stratum corneum with its horny and lipid layers. Stratum granulosum showed normal fibrillar content and keratohyalin granules. Keratinocytes of stratum spinosum revealed euchromatic nuclei with prominent nucleoli and joined by desmosomes. Stratum basale cells had basal oval nuclei arranged on the basement membrane (Figure 5A). However, neonates of the constant light group revealed thin stratum corneum and few keratohyalin granules in the stratum granulosum. Keratinocytes of stratum spinosum were flattened with irregular nuclear membrane. Stratum basale cells showed heterochromatic nuclei. Wide intercellular spaces and disrupted desmosomal junctions were observed (Figure 5B).

**Figure 6:**
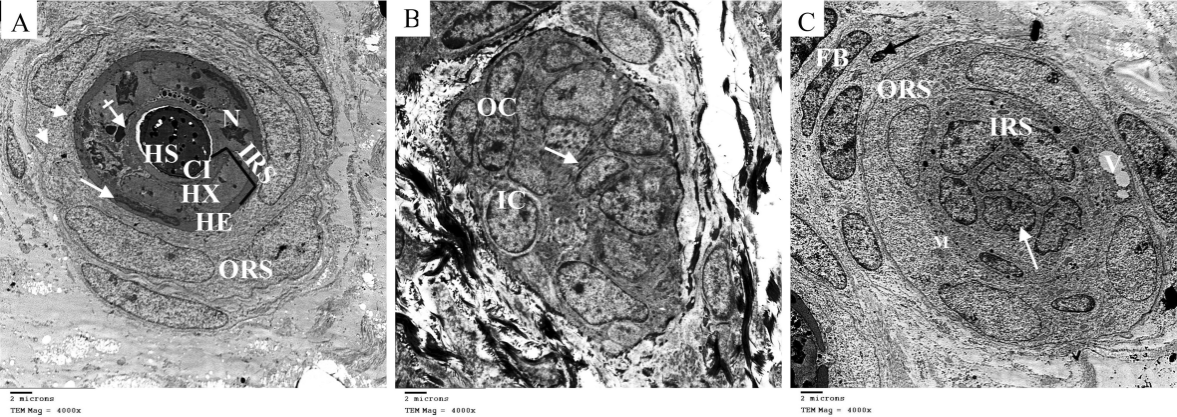
Electron micrographs of hair follicles of neonate rabbits (×4000, scale bar 2 μm). **A**, Control group showing mostly terminal stage hair follicle with hair shaft (HS) in the center surrounded by the three layers of inner root sheath (IRS) forming its cuticle (CI), Huxley's layer (HX) with its degenerated nucleus (N) and trichohyalin granules (crossed arrow), and cornified Henle's Layer (HE) with degradation of its nuclei (white arrow), surrounded by the outer root sheath (ORS) with intact mitochondria (arrow-head). **B** and **C**, Constant light group showing: **B**, immature hair follicle (mostly stage 2) before papilla intrusion formed of inner cells (IC) and outer cells (OC). Notice the tonofilament-like structures (arrow) formed by inner cells; **C**, mostly fourth stage hair follicle formed by inner root sheath (IRS) with a shrunken indented nucleus (white arrow), outer root sheath (ORS) with vacuolation (V) and swollen mitochondria (M), and surrounding fibroblasts (FB) with some apoptotic nuclei (black arrow).

The ultrastructural study of fully differentiated hair follicles (mostly terminal stage) of control neonates showed central keratinized hair shaft surrounded by the three layers of inner root sheath. The cuticle layer, Huxley's layer contained trichohyalin granules and degenerated nuclei and the cornified Henle's layer contained degenerated nucleus. The outer root sheath showed keratinocytes with intact mitochondria (Figure 6A). On the other hand, neonates of the constant light group revealed hair follicles in earlier stages and immature hair follicles (mostly second stage) formed by two layers of cells. The inner cells produced tonofilament-like structures (Figure 6B). Another hair follicle (mostly fourth stage) formed of undifferentiated inner root with some indented shrunken nuclei and outer root sheath with some vacuolation and swollen mitochondria. The hair follicle was surrounded by fibroblasts with some apoptotic nuclei (Figure 6C).

**Figure 7:**
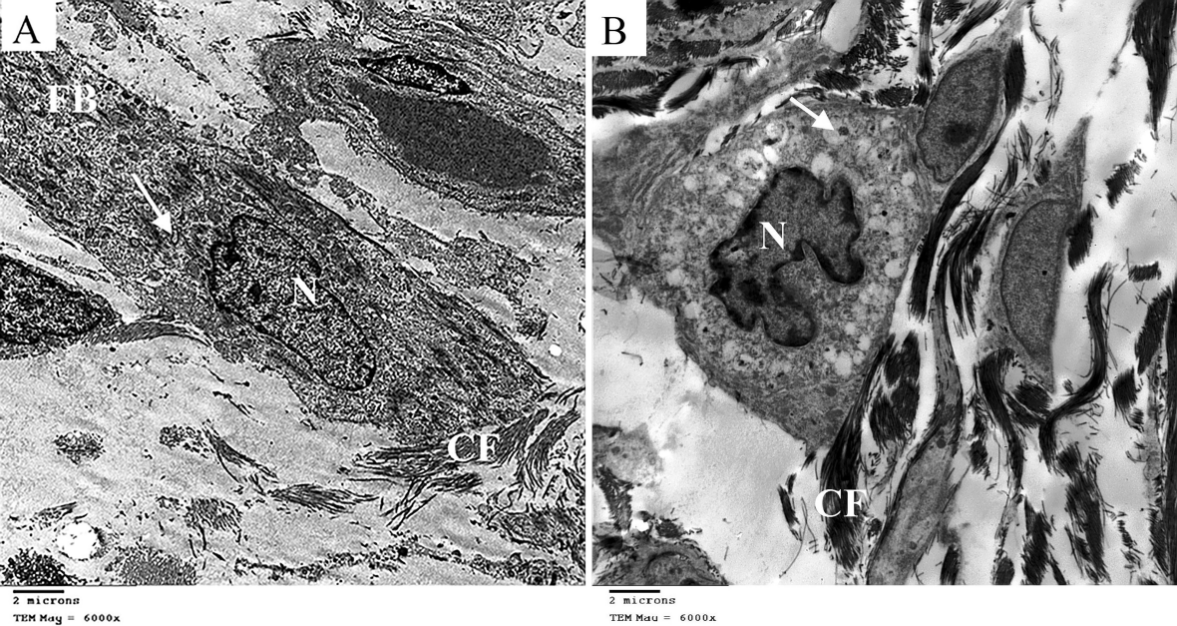
Electron micrographs of fibroblasts in the dermis of neonate rabbits (×6000, scale bar 2 μm). **A**, Control group showing dermal fibroblast (FB) with elongated nucleus (N) and rough endoplasmic reticulum (arrow) surrounded by collagen fibers (CF). **B**, Constant light group showing dermal fibroblast having folded nucleus with prominent nucleolus (N) and dilated cisternae of rough endoplasmic reticulum with electron dense material (arrow) surrounded by electron dense collagen fibers (CF).

The ultrastructural study of control neonatal dermis showed fibroblasts with characteristic elongated nucleus and abundant rough endoplasmic reticulum surrounded by collagen fibers (Figure 7A). However, neonates of the constant light group showed some dermal fibroblasts having folded nucleus with prominent nucleolus and dilated cisternae of rough endoplasmic reticulum containing electron dense material in its cytoplasm and surrounded by electron dense collagen fibers (Figure 7B).

## Discussion

The findings of our study suggested that circulating maternal nocturnal melatonin levels progressively increased during pregnancy in the control group with the maximum level at the 30th day of pregnancy. The constant light group showed significant reduction in maternal nocturnal melatonin levels compared to the control group. It is well established that rabbit pineal melatonin secretion shows a diurnal rhythm with maximum values at night (18). Variation of serum melatonin levels during pregnancy was also detected in humans (19) and rats (20) with higher values toward the end of pregnancy probably helping the fetus for birth. Exposure to constant light markedly reduced the maternal melatonin as reported in Sprague-Dawley rats (21) and capuchin monkeys (22).

The high serum maternal melatonin near the end of pregnancy can be explained on the basis of increased activity of maternal pineal gland in response to placental substances such as vasoactive intestinal polypeptide, testosterone, or neuropeptide Y (20).

The detected decreased serum maternal melatonin in the constant light group can be explained by the deactivation of N-acetyltransferase, which is considered one of the main enzymes for melatonin synthesis in the pineal gland (23). Moreover, Tan et al. (24) reported that under constant light conditions, pinealocytes, main cells that synthesize melatonin, showed degenerative changes in the form of swollen mitochondria with a rarified matrix and decreased cristae.

In the current study, the maternal nocturnal corticosterone levels showed no significant difference between groups. This finding suggested that exposure of constant light did not affect corticosterone levels in pregnancy. A similar finding was reported by Mendez et al. (21) in pregnant rats. Moreover, dim light at night did not alter corticosterone concentrations in mice (25). Fischman et al. (26) stated that rats maintained in constant light for 10 days showed adrenal insensitivity to the circulating ACTH.

On the other hand, Coomans et al. (27) concluded that exposure to constant light reduces corticosterone concentrations in mice. However, Milosević et al. (28) concluded that corticosterone levels increased in female rats chronically exposed to constant light. These widespread variations of the effect of constant light on glucocorticoids in different species may be explained by differences in the intensity, duration, and circadian phase of light exposure (29).

Therefore, in the current study, the maternal exposure to constant light with the consequent maternal chronodisruption and the associated reduction in maternal nocturnal melatonin level directly induced structural and ultrastructural alterations in neonate skin and not through a maternal stress effect.

The current study showed a marked reduction in the number of hair follicles that appeared at delayed stages of morphogenesis in neonates exposed to constant light compared with the control group. Moreover, thinning of stratum corneum and flattened keratinocytes of stratum spinosum were observed in the constant light group by TEM. Furthermore, morphometric measurements revealed a highly significant reduction in epidermal thickness in neonates of the constant light group. These findings can be related to the decreased maternal melatonin levels, which negatively affected the neonatal skin. Similar results were found in a rat model of pinealectomy by Eşrefoğlu et al. (30) who observed decreased epidermal thickness and hair follicle number.

Melatonin binding sites and cytoplasmic and nuclear receptors were detected in the skin, indicating that melatonin can control hair growth, inhibit hair follicle melanogenesis, and affect epidermal keratinocytes proliferation (31).

In addition, the reduced epidermal thickness of constant light neonates can be due to the significant decrease of PCNA immunopositive basal cells in this group as compared to control ones. Electron microscopy results showed stratum basale with heterochromatic nuclei, disrupted desmosomal junctions, and disorganized tonofilaments. These findings confirmed reduced cell proliferation and expanded turnover time according to Slominski et al. (31). In this context, Fujioka et al. (32) observed significantly decreased BrdU immunopositive cells, a marker of proliferating cells, in the hippocampus of constant light exposed mice.

Our electron microscopy results showed increased inter-cellular spaces with disrupted desmosomes in neonate skin of the constant light group. Desmosomes are intercellular junctions that strongly adhere the cells. Desmosomal disruption can alter tissue integrity and disturb intracellular signal transduction pathways, which interfere with cell proliferation, differentiation, and morphogenesis during embryonic development (33).

Nixon et al. (34) reported that melatonin may enhance the growing cycle of the hair follicle of New Zealand goats. These effects were considered to be due to melatonin stimulation of DNA synthesis in both epidermal and follicular keratinocytes (35).

In our model of maternal chronodisruption, TEM study of constant light neonates revealed some fibroblasts with folded nucleus and numerous rough endoplasmic reticulum with dilated cisternae containing electron-dense material. According to Wang et al. (36), this can be explained as the active state of protein synthesis. This was confirmed by our morphometric study of Masson's trichrome stained sections, which revealed significantly increased collagen fibers area percentage in neonates of the constant light group compared to the control ones. Drobnik and Dabrowski (37) observed increased collagen in both intact skins as well as in wound following pinealectomy. Taken together, melatonin has a key role in collagen homeostasis and its deficiency favors excess collagen formation.

Permanent light increases the lipoperoxide levels and reduces the antioxidative stress biomarkers such as reduced glutathione, glutathione peroxidase, and glutathione reductase (38). In addition, pregnancy is a physiological state accompanied by increased oxygen demand and increased reactive oxygen species production (39). Moreover, an *in vitro* study reported that melatonin use reduced the oxidative stress and apoptosis in the development of mammalian embryos (40). We hypothesized that the increased collagen, the observed apoptotic nuclei, the cytoplasmic vacuolation, and the mitochondria with altered morphology in neonates of the constant light group could be the results of associated oxidative stress and melatonin deficiency.

### Conclusions

The present results provided the first evidence of the impact of exposure to constant light during pregnancy and consequent maternal melatonin reduction on the neonatal New Zealand rabbit skin. Our findings revealed marked reduction in epidermal thickness and hair follicle number and increased collagen area percentage. Moreover, maternal exposure to constant light negatively affected the proliferative activity of keratinocytes. Therefore, a normal maternal circadian rhythm is a key factor for the integrity of neonatal skin structure.
